# Supplementary material for: High-Efficiency Oxygen Reduction Reaction Revived from Walnut Shell
Source: Molecules. 2023 Feb 22;28(5):2072. doi: 10.3390/molecules28052072 (PMC10003918; doi:10.3390/molecules28052072)
Supplement: Supplementary file 1 [file molecules-28-02072-s001.zip › molecules-2154682-supplementary.pdf]

Supplementary Material

High-Efficiency Oxygen Reduction Reaction Revived from Walnut Shell

Lei Yan [1], Yuchen Liu [1] and Junhua Hou [1,2,3,*]

[1] School of Physics and Information Engineering, Shanxi Normal University, No.339 Taiyu Road, Xiaodian District, Taiyuan 030031, China
[2] Extreme Optical Collaborative Innovation Center, Shanxi University, No. 92, Wucheng Road, Xiaodian District, Taiyuan 030006, China
[3] Modern College of Humanities and Sciences, Shanxi Normal University, No.501 Binhe West Road, Yaodu District, Linfen 041000, China
* Correspondence: jhhou@sxnu.edu.cn; Tel.: +86-13203574868

1. Experimental Section

1.1. Synthesis of Biomass Nanoparticles

Brush the walnut shell to clean the surface dirt， They were then immersed in deionized water and placed in an ultrasonic cleaner for an hour， Bake in the oven at 80 °C for 24 hours， You get dehydrated walnut shells， Then crush with crusher 32000 R/min, 10 minutes to get uniform 100 mesh walnut shell powder， Name it HTKC (HTKC stands for walnut shell, the same below).Put some more urea (white granules) into the crucible,Seal with aluminum and place in a tube furnace,The heating rate is 5°C/min,The temperature was set at 550 °C and annealed for 4 hours,A light yellow powder was obtained by full grinding in agate mortar,Name it G - (G - stands for urea after treatment, same as below),Mix walnut shells with G - in a ratio of 1:5, grind, pour in 30 ml deionized water,,And the uniform solution was obtained by ultrasonic 30 min in the ultrasonic instrument.The mixed solution was put into the reaction kettle and hydrothermal at 180°C for 15 h in the oven,Samples were washed and dried.In the atmosphere of nitrogen, the temperature was raised to 900°C at 5°C/min in the tubular furnace, and the temperature was held for 4 h,After cooling to room temperature, take out the black solid powder, after grinding, pickling once, washing to neutral, ethanol wash once.The powder was dried in an oven at 80 ° C to obtain a black powder denoted as NSCL-900. For comparison, it is denoted as NS-900 without urea doping, and it is denoted as NS-900 when urea is directly put into the reaction kettle with walnut shell without pyrolysis.

1.2. Evaluation of the Electrocatalytic Activity toward ORR

To prepare the electrode for ORR measurements, some GWS-X nanoparticles were dispersed in the solution of 50 Nafion solution, 250 isopropanol, and 700 deionized water were mixed in a 1.5 centrifuge tube and sonicated for 1 hour using a sonicator to form a catalyst suspension. Finally, take 10 droplets of the catalyst suspension on the surface of the electrode and wait for it to dry naturally (the average catalyst loading is 0.25).

The electrocatalytic activity was measured in a 0.1 M oxygen saturated KOH solution using an electrochemical workstation (CHI 760E, CH Instruments Inc., Shanghai, China) in a standard three electrode system. The three-electrode system was a catalyst-modified glass rotating disc electrode (GC-RDE) as the working electrode, with platinum wire and a 3 M/L KCl solution The Ag/AgCl electrodes are the counter electrode and the reference electrode. All measured potentials are referred to as reversible hydrogen electrodes (RHE) in 0.1 M KOH by RHE calibration, as shown in the following equation:

$$E_{RHE} = E_{Ag/AgCl} + 0.9762V \quad \text{(S1)}$$

Linear sweep voltammetry (LSV) was carried out at a scan rate of 1.0 mV s-1 for the whole polarization curves.

Table S1. The content of C, N, and O elements and N configuration calculated of NSCL-900, NS-900 and NNS-900 from elemental analysis and XPS.

Samples	Elemental content (at. %)			N configuration (%)			
	C 1s	N 1s	O 1 s	pyridinic-N	pyrrolic-N	graphitic-N	oxidized-N
NSCL-900	83.02	3.57	7.41	30.99	35.22	29.23	4.57
NS-900	90.15	2.33	7.52	28.94	32.15	29.90	8.68
NNS-900	93.25	0.00	6.75	0.00	0.00	0.00	0.00

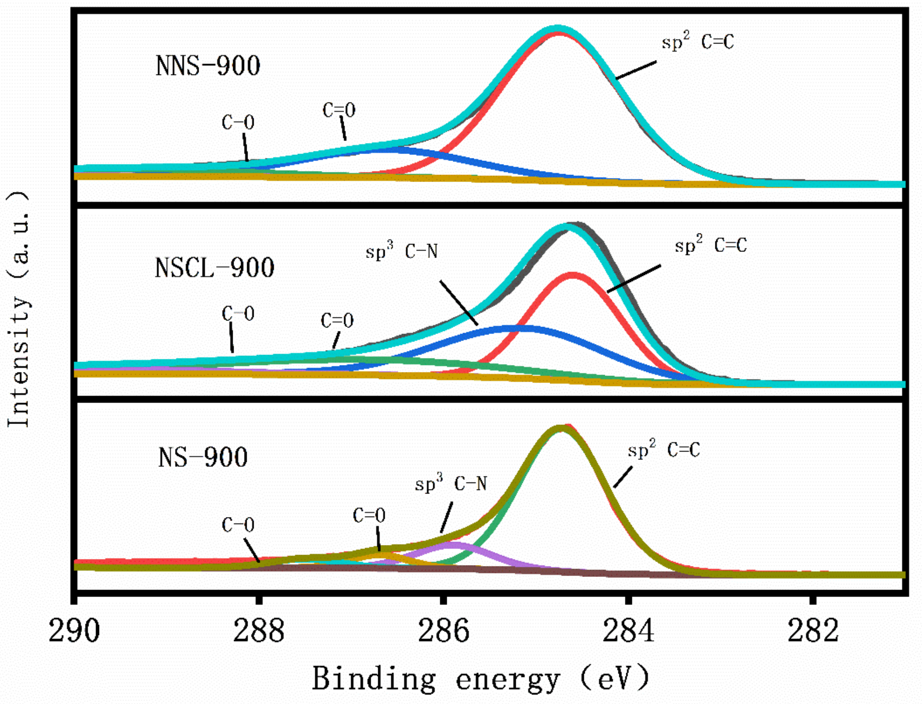

Figure S1. NNS-900, NSCL-900, NS-900 C1s spectrogram.
